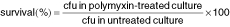# Acid pH activation of the PmrA/PmrB two-component regulatory system of *Salmonella enterica*

**DOI:** 10.1111/j.1365-2958.2006.05512.x

**Published:** 2007-01

**Authors:** J Christian Perez, Eduardo A Groisman

**Affiliations:** 1 Program in Molecular Genetics, Howard Hughes Medical Institute, Washington University School of Medicine Campus Box 8230, 660 S. Euclid Ave., St Louis, MO 63110, USA; 2 Department of Molecular Microbiology, Howard Hughes Medical Institute, Washington University School of Medicine Campus Box 8230, 660 S. Euclid Ave., St Louis, MO 63110, USA

## Abstract

Acid pH often triggers changes in gene expression. However, little is known about the identity of the gene products that sense fluctuations in extracytoplasmic pH. The Gram-negative pathogen *Salmonella enterica* serovar Typhimurium experiences a number of acidic environments both inside and outside animal hosts. Growth in mild acid (pH 5.8) promotes transcription of genes activated by the response regulator PmrA, but the signalling pathway(s) that mediates this response has thus far remained unexplored. Here we report that this activation requires both PmrA's cognate sensor kinase PmrB, which had been previously shown to respond to Fe^3+^ and Al^3+^, and PmrA's post-translational activator PmrD. Substitution of a conserved histidine or of either one of four conserved glutamic acid residues in the periplasmic domain of PmrB severely decreased or abolished the mild acid-promoted transcription of PmrA-activated genes. The PmrA/PmrB system controls lipopolysaccharide modifications mediating resistance to the antibiotic polymyxin B. Wild-type *Salmonella* grown at pH 5.8 were > 100 000-fold more resistant to polymyxin B than organisms grown at pH 7.7. Our results suggest that protonation of the PmrB periplasmic histidine and/or of the glutamic acid residues activate the PmrA protein, and that mild acid promotes cellular changes resulting in polymyxin B resistance.

## Introduction

Free-living organisms often encounter wide variations in the pH of their surroundings. Thus, pH may act as a signal that triggers cellular responses designed to cope with a new environment. The Gram-negative bacterium *Salmonella enterica* serovar Typhimurium, for example, experiences a number of acidic environments both inside and outside animal hosts. During infection of a mammalian host, *Salmonella* is exposed to severe acidity in the stomach (Rychlik and Barrow, 2005) and mild acidification in the endocytic vacuoles of intestinal epithelia and macrophages (Brumell and Grinstein, 2004). Moreover, *Salmonella* has been recovered from soil and water (Winfield and Groisman, 2003) where the pH can be significantly low. While growth in acidic conditions has been shown to promote changes in the gene expression profiles of several bacterial species (Tucker *et al*., 2002; McGowan *et al*., 2003; Weinrick *et al*., 2004; Leaphart *et al*., 2006), less is known about the identity of the molecule(s) that sense extracytoplasmic fluctuations in pH and the mechanisms by which such sensors promote changes in gene expression.

Previous studies have revealed that *Salmonella* responds to acidic challenges through an adaptive system called the acid tolerance response in which adaptation to mild acid conditions enables the organism to survive periods of severe acid stress (Foster and Hall, 1990; Foster, 1995). The acid tolerance response of *Salmonella* results in the synthesis of over 50 acid shock proteins (Bearson *et al*., 1998) that are likely to function primarily when variations in internal pH occur, i.e. when *Salmonella* experiences severe acidic conditions (pH ∼3) (Foster, 2004).

Growth of *Salmonella* in mild acid (pH 5.8) also promotes transcription of genes regulated by the response regulator PmrA (Soncini and Groisman, 1996). The expression of these genes has been shown to be dispensable for the acid tolerance response (Bearson *et al*., 1998) which suggests that there are still uncharacterized cellular function(s) that *Salmonella* needs to regulate in acidic environments. The PmrA protein and its cognate sensor kinase PmrB form a two-component regulatory system that is required for virulence in mice (Gunn *et al*., 2000), infection of chicken macrophages (Zhao *et al*., 2002), growth in soil (Chamnongpol *et al*., 2002), resistance to the cationic peptide antibiotic polymyxin B (Roland *et al*., 1993) and resistance to Fe^3+^- (Wosten *et al*., 2000) and Al^3+^-mediated killing (Nishino *et al*., 2006). The PmrA-regulated products characterized thus far mediate modifications to the various components of the lipopolysacharide (LPS) structure including the lipid A (Gunn *et al*., 1998; Trent *et al*., 2001; Zhou *et al*., 2001; Breazeale *et al*., 2003; Lee *et al*., 2004), the core region (Nishino *et al*., 2006) and the O-antigen (Delgado *et al*., 2006). While other PmrA-regulated genes have been identified (Marchal *et al*., 2004; Tamayo *et al*., 2005), their biochemical activities and the role(s) that they play in *Salmonella*'s life remain unknown.

**Fig. 1:**
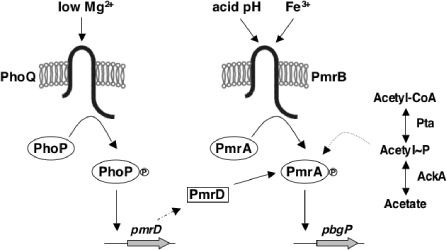
Model depicting the pathways leading to activation of the PmrA protein. Fe^3+^ and acid pH are sensed by the PmrB protein, which promotes phosphorylation of PmrA, resulting in transcription of PmrA-activated genes. The PhoQ protein senses extracellular Mg^2+^. In low Mg^2+^, PhoQ promotes phosphorylation of PhoP and transcription of *pmrD*. The PmrD protein binds to the phosphorylated form of PmrA protecting it from dephosphorylation by PmrB. Acetyl phosphate, which is synthesized by the enzymes phosphotransacetylase (Pta) and acetate kinase (AckA), activates PmrA in a strain deleted for the *pmrB* gene.

Besides mild acid pH, two other stimuli are known to promote expression of PmrA-activated genes: (i) submillimolar levels of extracellular Fe^3+^ or Al^3+^, which are directly sensed by the PmrB protein (Wosten *et al*., 2000), and (ii) low concentrations of extracellular Mg^2+^ (Soncini and Groisman, 1996) (Fig. 1). The low Mg^2+^ activation of the PmrA protein requires PhoQ, a protein that senses extracellular Mg^2+^ levels (Vescovi *et al*., 1996), PhoQ's cognate regulator PhoP, and the PhoP-activated protein PmrD (Kox *et al*., 2000; Kato and Groisman, 2004). PmrD binds to the phosphorylated form of PmrA protecting it from dephosphorylation by PmrB (Kato and Groisman, 2004). Here we show that PmrA's cognate sensor kinase PmrB is required for responding to external changes in pH through a mechanism that requires a histidine and several glutamic acid residues located in its periplasmic domain, as well as the post-translational activator PmrD protein.

## Results

### Mild acid pH induces transcription of PmrA-regulated genes

**Fig. 2:**
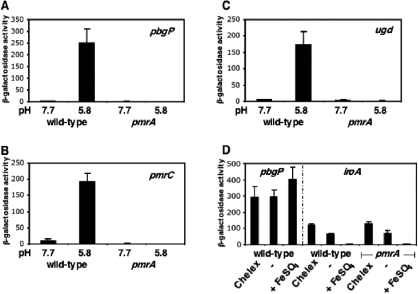
Mild acid pH promotes transcription of PmrA-regulated genes. A–C. β-Galactosidase activity (Miller units) expressed by strains harbouring chromosomal *lac* transcriptional fusions to the PmrA-activated *pbgP* (EG9241, EG9681) (A), *pmrC* (EG9279, EG9687) (B) and *ugd* (EG9524, EG9674) (C) genes. Strain numbers are indicated in parenthesis, with the first one corresponding to the *pmrA*^+^ and the second to the *pmrA* background. Expression was investigated in wild-type and *pmrA* backgrounds following growth in N-minimal medium pH 7.7 or 5.8 as described under *Experimental procedures*. Shown are the mean values and standard deviations of three independent experiments performed in duplicate. D. β-Galactosidase activity (Miller units) expressed by strains harbouring chromosomal *lac* transcriptional fusions to the PmrA-activated *pbgP* (EG9241) and iron-repressed *iroA* (EG12735, EG12737) genes. Cells were grown in Chelex-treated or untreated N-minimal medium pH 5.8. FeSO_4_ (100 μM) was added to the Chelex-treated medium where indicated. Shown are the mean values and standard deviations of three independent experiments performed in duplicate.

To examine the mild acid pH induction of PmrA-activated genes, we grew *Salmonella* cells harbouring chromosomal *lacZYA* transcriptional fusions to the PmrA-regulated genes *pbgP*, *pmrC* and *ugd* (Wosten and Groisman, 1999) in N-minimal media buffered at pH 5.8 or 7.7. This medium lacked Fe^3+^ or Al^3+^, the only known PmrB ligands (Wosten *et al*., 2000), and contained 10 mM MgCl_2_, which represses expression of PmrA-activated genes (Soncini and Groisman, 1996; Kox *et al*., 2000). All three genes were expressed when cells were grown in media buffered at pH 5.8 but not at pH 7.7 (Fig. 2A–C), in agreement with previous results (Soncini and Groisman, 1996). A similar induction of *pbgP* transcription was found when MES was used as the buffering agent in the media at pH 5.8 instead of Bis-Tris (data not shown), indicating that the mild acid effect on gene expression was not due to a particular buffering system.

The transcriptional activation of PmrA-regulated genes taking place at pH 5.8 could be due to trace amounts of metals such as Fe^3+^, which is more soluble at acidic pH. To rule out this possibility, we treated the culture medium with Chelex 100 resin, an agent known to chelate polyvalent metal ions that does not affect *Salmonella* growth. We determined that Chelex 100 was effective at chelating iron because expression of the *pmrA*-independent iron-repressed *iroA* gene (Hall and Foster, 1996) was induced to higher levels in cultures treated with Chelex 100 (Fig. 2D). Expression of *pbgP* was still induced when *Salmonella* was grown in the Chelex-treated medium (Fig. 2D) or in media containing the specific Fe^3+^ chelator deferoxamine mesylate (data not shown) supporting the notion that mild acid pH is responsible for the observed induction.

We determined that the regulatory protein PmrA is required for the transcriptional activation in response to mild acid pH because there was no induction of the three investigated genes in a *pmrA* mutant (Fig. 2A–C). Moreover, a mutant expressing a derivative of the PmrA protein that cannot be phosphorylated due to substitution of the putative phosphorylation residue aspartate 51 by alanine (Kato and Groisman, 2004) completely failed to promote transcription of PmrA-activated genes in response to pH 5.8, in a similar fashion to the *pmrA* strain (A. Kato and E.A. Groisman, unpubl. results). From these results we conclude that *Salmonella* harbours a signalling pathway that responds to mild acid pH by activating the PmrA protein through phosphorylation.

### The PmrB protein is necessary for the mild acid activation of PmrA

**Fig. 3:**
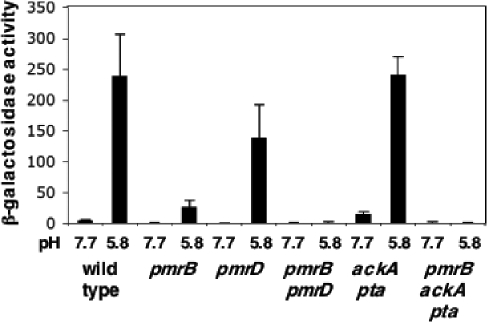
The PmrA-cognate sensor PmrB is required to activate the PmrA-regulated gene *pbgP* in response to mild acid pH. β-Galactosidase activity (Miller units) expressed by strains harbouring a chromosomal *lac* transcriptional fusion to the *pbgP* gene. Expression was investigated in wild-type (EG9241), *pmrB* (EG16704) and *pmrD* (EG11775) mutant, *pmrB pmrD* (EG12060) and *ackA pta* (EG16450) double mutant and the *pmrB ackA pta* triple mutant (EG16706) backgrounds. Cells were grown in N-minimal medium pH 7.7 or 5.8 as described under *Experimental procedures*. Shown are the mean values and standard deviations of three independent experiments performed in duplicate.

The PmrB protein is necessary for activation of the PmrA protein in low Mg^2+^ (Kox *et al*., 2000; Kato and Groisman, 2004) and in the presence of Fe^3+^ (Wosten *et al*., 2000), consistent with the notion that PmrB is the major phosphodonor for PmrA. We investigated whether PmrB was also required for the pH-dependent induction of *pbgP*, which was chosen as a prototypical PmrA-activated gene because the PmrA protein binds to the *pbgP* promoter *in vitro* (Wosten and Groisman, 1999) and *in vivo* (Shin and Groisman, 2005). Thus, we determined the β-galactosidase activity of isogenic *pmrB* strains harbouring a chromosomal *pbgP–lac* transcriptional fusion: expression was approximately sixfold lower in a *pmrB* mutant than in the *pmrB*^+^ strain following growth at pH 5.8 (Fig. 3), indicating that a functional *pmrB* gene is necessary for a normal response to mild acid pH.

There was residual *pbgP* expression in the *pmrB* mutant induced with mild acid pH (Fig. 3), which was in contrast to the absence of *pbgP* transcription in the *pmrA* mutant (Fig. 2). This suggested that PmrA could become phosphorylated from another phosphodonor(s) when PmrB is not present. We considered the possibility of PmrA being phosphorylated from acetyl phosphate because acetyl phosphate has been shown to serve as phosphoryl donor to several response regulators when their cognate sensors are absent (see Wolfe, 2005 for a review). Consistent with this notion, *pbgP* transcription was abrogated in the *pmrB* mutant upon deletion of the *pta* and *ackA* genes (Fig. 3), which encode the two enzymes that are required for the production of acetyl phosphate (Wolfe, 2005) (Fig. 1). In contrast, a strain lacking the ability to synthesize acetyl phosphate but with a functional *pmrB* gene exhibited wild-type *pbgP* expression levels (Fig. 3), implying that under normal conditions (i.e. when a functional *pmrB* gene is present) acetyl phosphate does not contribute to PmrA phosphorylation.

### The PmrD protein is necessary for normal PmrA activation at pH 5.8

The PhoP-activated PmrD protein favours the phosphorylated state of the PmrA protein (Fig. 1) (Kato and Groisman, 2004). Thus, we tested the possibility of PmrD participating in the PmrA-dependent response to acidic conditions, and thus contributing to the *pbgP* transcription remaining in a *pmrB* mutant. Expression of the *pbgP* gene was abolished in a *pmrB pmrD* double mutant (Fig. 3) indicating that both genes are necessary to activate PmrA under acidic conditions. In contrast to the phenotype of the *pta ackA* double mutant, *pbgP* transcription was reduced in the *pmrD* mutant (Fig. 3). These results imply that the *pmrD* gene was being expressed even though the media contained 10 mM MgCl_2_, a concentration known to repress transcription of PhoP-activated genes (Soncini *et al*., 1996).

**Fig. 4:**
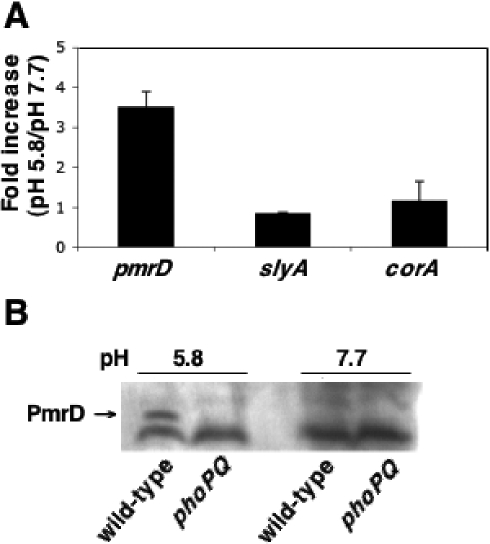
Expression of the *pmrD* gene is promoted under mild acid pH. A. RNA levels of transcripts corresponding to the PhoP-activated *pmrD* and *slyA* genes and to the PhoP-independent *corA* gene as determined by quantitative real-time PCR. Shown are the mean values and standard deviations of three independent experiments. B. Western blot analysis of crude bacterial extracts prepared from wild-type (14028s) or *phoPQ* (EG15598) cells grown in N-minimal medium at pH 5.8 or 7.7 as described under *Experimental procedures*. The upper band corresponds to PmrD. The lower band is a non-specific cross-reactive product that indicates equal protein loading across the lanes.

We examined transcription of the *pmrD* gene using RNA isolated from organisms grown at pH 5.8 or 7.7. Growth at pH 5.8 resulted in *pmrD* transcript levels that were ∼3.5-fold higher than in organisms grown at pH 7.7 (Fig. 4A). This acid pH-promoted increase appears to be specific to a subset of PhoP-activated genes (our unpublished results) that includes *pmrD* because expression of the PhoP-regulated *slyA* gene and the PhoP-independent *corA* gene was not affected by the pH of the medium (Fig. 4A). In agreement with the gene transcription data, Western blot analysis of crude extracts using anti-PmrD antibodies showed that the PmrD protein was produced in cells grown in N-minimal medium pH 5.8 and 10 mM MgCl_2_ but not in cells grown in the same medium buffered at pH 7.7 (Fig. 4B). The acid-promoted expression of the PmrD protein was *phoPQ*-dependent, which is in agreement with the fact that PhoP is the only known direct transcriptional activator of *pmrD* (Kox *et al*., 2000).

### Conserved histidine and glutamic acid residues in the periplasmic domain of PmrB are required for signalling in response to mild acid pH

**Fig. 5:**
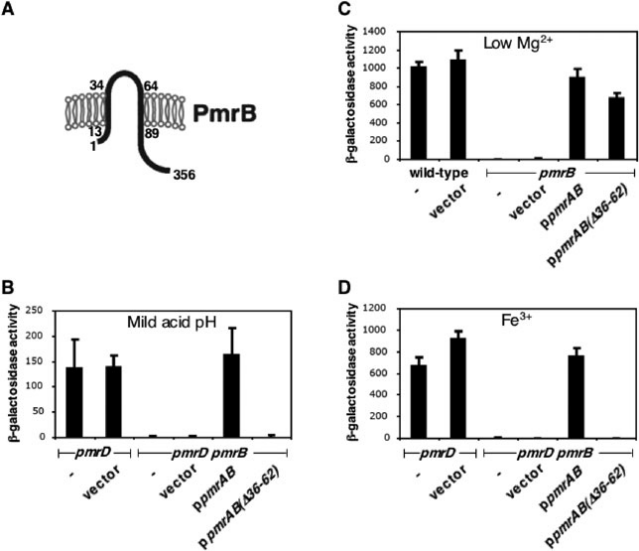
The periplasmic domain of the PmrB protein is required for responding to mild acid pH. A. Predicted topology of the sensor kinase PmrB in the inner membrane. Numbers indicate amino acid positions. B–D. β-Galactosidase activity (Miller units) expressed by wild type (EG9241), *pmrB* (EG10065) and *pmrD pmrB* (EG12060) mutant strains harbouring a *lac* transcriptional fusion to *pbgP* and either the plasmid vector pUHE21*lac*I^q^, plasmid p*pmrAB* expressing the wild-type *pmrAB* genes or plasmid p*pmrAB*(Δ*36-62)* expressing the wild-type PmrA protein and a PmrB protein deleted for 26 of its 31 periplasmic domain residues. Cells were grown in N-minimal medium containing 10 mM MgCl_2_, pH 5.8 (B), 10 μM MgCl_2_, pH 7.7 (C) or 10 μM MgCl_2_, 100 μM FeSO_4_, pH 7.7 (D). The β-galactosidase activity in all strains grown under non-inducing conditions, i.e. N-minimal medium containing 10 mM MgCl_2_, pH 7.7, was undetectable. Shown are the mean values and standard deviations of three independent experiments performed in duplicate.

The results described above established that PmrB is required for activation of PmrA in response to mild acid pH. This could be because PmrB is directly involved in sensing extracytoplasmic pH in a way analogous to its sensing of Fe^3+^ and Al^3+^ (Wosten *et al*., 2000), or because PmrB plays an indirect role in its capacity of main (if not sole) phosphodonor for PmrA. In fact, PmrB is required for the activation of PmrA-regulated genes in response to the low Mg^2+^ signal, which is sensed by the PhoQ protein (Kato and Groisman, 2004) (Fig. 1). Thus, we reasoned that if PmrB senses extracytoplasmic pH directly, its periplasmic domain (Fig. 5A) was likely to be required for the response to this signal. To examine this hypothesis, we tested a *Salmonella* strain with a chromosomal *pbgP–lac* fusion, deleted for the chromosomal copy of the *pmrB* gene and harbouring a plasmid expressing a PmrB protein lacking its periplasmic domain for its ability to promote *pbgP* expression in response to different signals. There was no *pbgP* expression in cells grown at pH 5.8 (Fig. 5B) or in the presence of Fe^3+^ (Fig. 5D), which is in contrast to the normal activation in response to low Mg^2+^ (Fig. 5C). Together, these results argue in favour of the notion that PmrB senses extracellular pH besides its previously described ligands Fe^3+^ and Al^3+^ (Wosten *et al*., 2000).

**Fig. 6:**
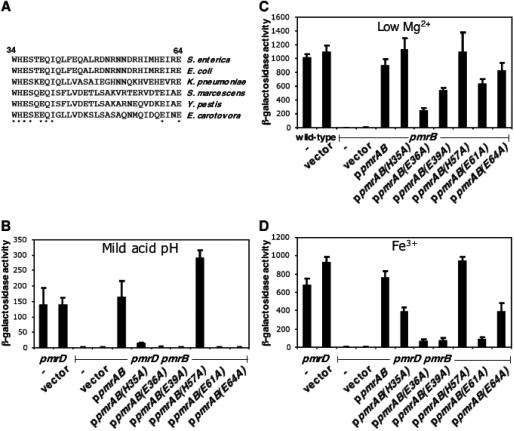
Conserved histidine and glutamic acid residues in the periplasmic domain of the PmrB protein are required for PmrA-mediated transcription in response to mild acid pH. A. Alignment of the amino acid sequences corresponding to the putative periplasmic domains of the PmrB proteins from *Salmonella enterica*, *Escherichia coli*, *Klebsiella pneumoniae*, *Serratia marcescens*, *Yersinia pestis* and *Erwinia carotovora*. Asterisks (*) denote residues conserved in all six species. B–D. β-Galactosidase activity (Miller units) expressed by wild-type (EG9241), *pmrB* (EG10065) and *pmrD pmrB* (EG12060) mutant strains harbouring a *lac* transcriptional fusion to *pbgP* and plasmid vector pUHE21*lac*I^q^, plasmid p*pmrAB* expressing the wild-type *pmrAB* genes, or plasmids in which the nucleotide sequence corresponding to periplasmic histidines and glutamates were mutated to alanine [p*pmrAB*(*H35A*), p*pmrAB*(*E36A)*, p*pmrAB*(*E39A)*, p*pmrAB*(*H57A)*, p*pmrAB*(*E61A)*, p*pmrAB*(*E64A)*]. Cells were grown in N-minimal medium containing 10 mM MgCl_2_, pH 5.8 (B), 10 μM MgCl_2_, pH 7.7 (C) or 10 μM MgCl_2_, 100 μM FeSO_4_, pH 7.7 (D). The β-galactosidase activity in all strains grown under non-inducing conditions, i.e. N-minimal medium containing 10 mM MgCl_2_, pH 7.7, was undetectable. Shown are the mean values and standard deviations of three independent experiments performed in duplicate.

An alignment of the amino acid sequences corresponding to the putative periplasmic domain of the PmrB proteins from six enteric species revealed that nine residues are highly conserved (Fig. 6A). Interestingly, one of these conserved residues was a histidine at position 35. Because the p*K*_a_ of free histidine is ∼6, the pH at which PmrA-activated genes are induced, we hypothesized that this residue might be required for pH sensing. To test this hypothesis, we constructed a plasmid that produced a PmrB protein containing a single histidine to alanine substitution at position 35. While this mutation severely diminished the ability of *Salmonella* to respond to mild acid pH, there still was some residual *pbgP* expression (Fig. 6B) suggesting that other residues might also be required for pH sensing. We considered the possibility that a second histidine at position 57 could be involved in sensing acid despite the fact that this residue was only partially conserved across species (Fig. 6A). However, the substitution of this residue by alanine had no effect on the response to mild acid pH (Fig. 6B).

Four of the nine conserved amino acids in the periplasmic domain of PmrB are glutamic acid residues, which also could be subjected to changes in protonation upon variations in the pH of their surroundings. Although the p*K*_a_ of free glutamic acid is ∼4, which is well below the range of pH at which PmrA-activated genes are induced, the folding of a protein can dramatically change the p*K*_a_ of its residues. For instance, the p*K*_a_ of one of the glutamic acid residues of the regulatory protein TraM is ∼7.7 (Lu *et al*., 2006). Therefore, we hypothesized that one or more of the glutamates might be required for pH sensing. To test this hypothesis, we used plasmids that produced PmrB proteins containing single-amino-acid replacements in the conserved glutamic acid residues. When either one of the four conserved glutamates was substituted by alanine *Salmonella* could no longer respond to mild acid pH (Fig. 6B). Strains expressing the mutant PmrB proteins could express *pbgP* normally in response to the low Mg^2+^ signal (Fig. 6C) (Wosten *et al*., 2000), indicating that mutations in residues of the periplasmic domain of PmrB do not impair the enzymatic activity of the cytoplasmic domain of the PmrB protein. These results indicate that the periplasmic glutamates are required for responding to mild acid pH.

### Mild acid pH induces resistance to the antimicrobial peptide polymyxin B

**Fig. 7:**
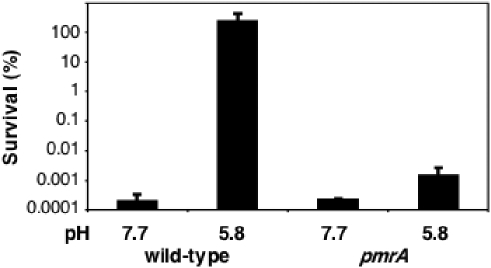
Mild acid pH induces resistance to the antimicrobial peptide polymyxin B. Per cent survival of wild-type (14028s) and *pmrA* (EG7139) strains after incubation with polymyxin B (1.5 μg ml^−1^). Cells were grown in N-minimal medium, pH 7.7 or 5.8, containing 10 mM MgCl_2_, before incubation with polymyxin B. Shown are the mean values and standard deviations of three independent experiments performed in duplicate.

What role could the mild acid pH-dependent activation of PmrA-regulated genes play in *Salmonella*'s lifestyle? Because the PmrA/PmrB system is required for resistance to the antimicrobial peptide polymyxin B (Roland *et al*., 1993), we hypothesized that mild acid pH could induce this resistance. In fact, the survival of wild-type cells to a challenge with polymyxin B was 100 000-fold higher when they were grown at pH 5.8 than when grown at pH 7.7 (Fig. 7). This resistance was PmrA-dependent because a strain deficient in the *pmrA* gene was approximately 100 000-fold more sensitive to polymyxin B than the wild-type strain when grown pH 5.8 (Fig. 7).

## Discussion

We have established that the sensor kinase PmrB is the primary sensor that activates the PmrA protein when *Salmonella* experiences mild acid pH, resulting in transcription of PmrA-activated genes (Fig. 1). That PmrB is likely to sense changes in pH directly is supported by three findings: (i) the mild acid pH-dependent activation of the PmrA-regulated gene *pbgP* was dramatically reduced in a strain lacking *pmrB* (Fig. 3), (ii) the periplasmic domain of PmrB was necessary for activation of *pbgP* under mild acid conditions (Fig. 5), and (iii) single amino acid substitutions in conserved histidine and glutamic acid residues located in the periplasmic domain of PmrB abolished its ability to stimulate *pbgP* transcription at pH 5.8 (Fig. 6). The periplasmic histidine and glutamates are conserved in the PmrB periplasmic domain of other enteric species, raising the possibility that the signalling pathway described in this article may be operating in other organisms in addition to *S. enterica*.

The requirement of periplasmic PmrB residues in the mild acid pH activation of PmrA-regulated genes suggests that this signalling pathway responds to changes in extracytoplasmic pH. Moreover, under the experimental conditions used in this study it is unlikely that the cytoplasmic pH varied significantly because: first, bacterial cells can maintain an internal pH of up to 2 units higher than the external pH (Foster, 2004); in fact, Slonczewski *et al*. (1981) determined that the intracellular pH in *Escherichia coli* cells was 7.4 even when the external pH was 5.5. Second, acid stress can become a severe challenge for bacterial cells when organic acids such as acetate or products of fermentation are present in the medium (Bearson *et al*., 1998); and in our experiments we used a non-fermentable sugar (glycerol) and inorganic acids which are not expected to cause such acid stress.

Structural changes driven by a relatively narrow variation in pH (1–2 units) have been reported for several cytosolic bacterial proteins (Tews *et al*., 2005; Lu *et al*., 2006). This is in contrast to the few membrane proteins (other than ion channels) that have been shown to respond to changes in extracellular pH of a similar magnitude. For example, the eukaryotic G-protein coupled receptor OGR1 is inactive at pH 7.8 and fully active at pH 6.8 suggesting that the pH sensing mechanism involves protonation of several extracytoplasmic histidines (Ludwig *et al*., 2003), which is in agreement with the p*K*_a_ of free histidine of ∼6. In the case of PmrB, a normal response to mild acid pH requires not only a periplasmic histidine but also several glutamic acid residues. Therefore, regulation of PmrB activity may involve protonation of one or more of these amino acids. Even though protonation of the glutamic acid residues may seem unlikely given the fact that the p*K*_a_ of free glutamic acid is ∼4, protein folding can change the p*K*_a_ of its residues (Tanford and Roxby, 1972). Indeed, the p*K*_a_ of one of the glutamic acid residues of the regulatory protein TraM is ∼7.7 in the folded protein (Lu *et al*., 2006). Therefore, it is plausible that protonation/deprotonation of one or more of the glutamic acids in the periplasmic domain of PmrB could occur at pH ∼5.8.

Integral membrane proteins that recognize signals in addition to extracytoplasmic pH, such as PmrB, have been identified both in prokaryotes and in eukaryotes. The CadC protein of *E. coli*, for example, is activated by exogenous lysine besides acid pH (Dell *et al*., 1994). Likewise, the human receptor OGR1 responds to both pH and sphingosylphosphorylcholine (Ludwig *et al*., 2003). The fact that the PmrB H35A and the E64A mutant proteins displayed partial activity in response to ferric iron but were severely impaired in their ability to respond to acid pH (compare Fig. 6B and D) supports the notion that these signals are sensed independently. Similarly, *cadC* mutants have been isolated that are impaired in the ability to sense only one of its two inducing signals (Dell *et al*., 1994). Furthermore, the ability to sense two different compounds has also recently been shown to be genetically distinguishable in the bacterial chemoreceptor Tcp (Iwama *et al*., 2006).

The PmrB protein plays the primary role in the pH-dependent activation of PmrA, but full activation also requires PmrD, the post-translational activator of the PmrA protein (Fig. 3). The levels of phosphorylated PmrA are determined by the balance of the autokinase + phosphotransferase activity of PmrB and PmrB's phosphatase activity towards phospho-PmrA. Thus, PmrD may be necessary to ensure that the amount of phosphorylated PmrA is such to promote transcription of its regulated genes. Consistent with its role in acid pH activation, expression of the *pmrD* gene was promoted in media of mild acid pH (Fig. 4). The mechanism(s) by which acid pH leads to an increase in the levels of the *pmrD* transcript, however, remains unclear. Although it has been suggested that the *Salmonella* PhoQ protein senses acid pH (Aranda *et al*., 1992) or responds to both pH and Mg^2+^ (Bearson *et al*., 1998), a direct role for PhoQ in responding to acid pH appears unlikely because not all PhoP-regulated genes are activated under these conditions, which is in contrast to low Mg^2+^ activating the whole PhoP regulon (see Groisman and Mouslim, 2006 for a review).

What role could the pH-dependent activation of PmrA-regulated genes play in *Salmonella*'s lifestyle? Because several PmrA-activated gene products are responsible for remodelling the LPS structure and these modifications are required for resistance to certain antimicrobial peptides and toxic metals, one possibility is that acidic environments provide a means to induce the cell envelope changes resulting in resistance. Indeed, when grown at pH 5.8 wild-type *Salmonella* were 100 000-fold more resistant to polymyxin B than when grown at pH 7.7 (Fig. 7). This may be particularly important for *Salmonella* living in soil due to the fact that the antimicrobial peptide polymyxin B is produced by the soil bacterium *Paenibacillus polymyxa* (Paulus and Gray, 1964) and because the solubility of metals such as Fe^3+^ increases in acid pH. On the other hand, although mild acid (pH 6.0) *per se*, i.e. even in the presence of high Mg^2+^, promotes LPS modifications (Gibbons *et al*., 2005), the low pH signal may also act synergistically with the low Mg^2+^ signal *in vivo* because Mg^2+^ deprivation alone is not sufficient to provide all the LPS modifications seen in *Salmonella* when present inside macrophages (Gibbons *et al*., 2005). Finally, while a role for the PmrA-dependent LPS modifications in the previously described acid tolerance response is unlikely because survival to acid stress (pH ∼3) was not reduced in cells deficient in *pmrA* (data not shown and Bearson *et al*., 1998), some of the PmrA-regulated genes to which no function has been ascribed yet could mediate other cellular responses to acid pH.

## Experimental procedures

### Bacterial strains, plasmids and growth conditions

Bacterial strains and plasmids used in this study are listed in Table 1. All *S. enterica* serovar Typhimurium strains are derived from wild-type 14028s and were constructed by phage P22-mediated transductions as described elsewhere (Davis *et al*., 1980). Bacteria were grown at 37°C in N-minimal media (Snavely *et al*., 1991) buffered in 50 mM Bis-Tris (or MES), pH 7.7 or 5.8, supplemented with 0.1% casamino acids, 38 mM glycerol and 10 μM or 10 mM MgCl_2_. When indicated, medium was treated overnight with Chelex 100 resin (Sigma) to chelate metal ions before using it for cell culture. Deferoxamine mesylate (Sigma) was used at a final concentration of 300 μM. FeSO_4_ was used at 100 μM. *E. coli* DH5α was used as the host for preparation of plasmid DNA. Ampicillin and kanamycin were used at 50 μg ml^−1^ and chloramphenicol was used at 20 μg ml^−1^.

**Table 1:** Bacterial strains and plasmids used in this study.

Strain or plasmid	Description	Reference or source
*S. enterica*		
14028s	Wild type	ATCC
EG7139	*pmrA*::Cm^R^	Soncini and Groisman (1996)
EG9241	*pbgP::*MudJ	Soncini *et al*. (1996)
EG9681	*pmrA*::Cm^R^*pbgP*::MudJ	Soncini and Groisman (1996)
EG9279	*pmrC*::MudJ	Soncini and Groisman (1996)
EG9687	*pmrA*::Cm^R^*pmrC*::MudJ	Soncini and Groisman (1996)
EG9524	*ugd*::MudJ	Vescovi *et al*. (1996)
EG9674	*pmrA*::Cm^R^*ugd*::MudJ	Soncini and Groisman (1996)
EG12735	*iroA1*::MudJ	Hall and Foster (1996)
EG12737	*pmrA*::Cm^R^*iroA1*::MudJ	*S.* Chamnongpol and E.A. Groisman (unpublished)
EG10065	*pmrB*::Cm^R^*pbgP*::MudJ	Kox *et al*. (2000)
EG11775	*pmrD*::Cm^R^*pbgP*::MudJ	Kox *et al*. (2000)
EG12060	*pmrB*::Cm^R^*pmrD*::Cm^R^*pbgP*::MudJ	Kox *et al*. (2000)
EG15598	Δ*phoP/phoQ*::Cm^R^	Shin and Groisman (2005)
EG16443	Δ*ackA/pta*::Cm^R^	This work
EG16450	Δ*ackA/pta*::Cm^R^*pbgP::*MudJ	This work
EG16704	Δ*pmrB pbgP::*MudJ	This work
EG16706	Δ*pmrB*Δ*ackA/pta*::Cm^R^*pbgP::*MudJ	This work
*E. coli*		
DH5α	F^–^*sup*E44 Δ*lac*U169 (φ80 *lacZ*ΔM15) *hsd*R17 *rec*A1 *end*A1 *gyr*A96 *thi*-1 *rel*A1	Hanahan (1983)
Plasmids		
pUHE21-2*lacI*^q^	rep_pMB1_ Ap^R^*lacI* ^q^	Soncini *et al*. (1995)
pEG9102	rep_pMB1_ Ap^R^*lacI* ^q^*pmrAB*	Soncini and Groisman (1996)
pUH*pmrAB(*Δ*36-62)*	rep_pMB1_ Ap^R^*lacI* ^q^*pmrAB (*Δ*36-62)*	Wosten et al. (2000)
pUH*pmrAB(H35A)*	rep_pMB1_ Ap^R^*lacI* ^q^*pmrAB (H35A)*	This work
pUH*pmrAB(E36A)*	rep_pMB1_ Ap^R^*lacI* ^q^*pmrAB (E36A)*	Wosten et al. (2000)
pUH*pmrAB(E39A)*	rep_pMB1_ Ap^R^*lacI* ^q^*pmrAB (E39A)*	Wosten et al. (2000)
pUH*pmrAB(H57A)*	rep_pMB1_ Ap^R^*lacI* ^q^*pmrAB (H57A)*	This work
pUH*pmrAB(E61A)*	rep_pMB1_ Ap^R^*lacI* ^q^*pmrAB (E61A)*	Wosten et al. (2000)
pUH*pmrAB(E64A)*	rep_pMB1_ Ap^R^*lacI* ^q^*pmrAB (E64A)*	Wosten et al. (2000)
pKD3	rep_R6Kγ_ Ap^R^ FRT Cm^R^ FRT	Datsenko and Wanner (2000)
pKD46	rep_pSC101_ts Ap^R^ p_*araBAD*_γβ exo	Datsenko and Wanner (2000)
pCP20	rep_pSC101_ts Ap^R^ Cm^R^*cl*857 λP_R_*flp*	Cherepanov and Wackernagel (1995)

### Construction of chromosomal gene deletion mutants and plasmids

Strain EG16443, which has a deletion of both the *ackA* and *pta* genes, was constructed by the one-step gene inactivation method (Datsenko and Wanner, 2000) as follows: a Cm^R^ cassette was amplified using primers 5956 (5′-CTGACGTTTTTTTAGCCACGTATCATAAATAGGTACTTCCGTGTAGGCTGGAGCTGCTTC-3′) and 5957 (5′-TTACTGCTGCTGCTGAGAAGCCTGGATCGCCGTCAGGGCGCATATGAATATCCTCCTTAG-3′) and pKD3 as template and recombined into the *ackA pta* region in strain 14028s. The structure of the generated mutant was verified by colony PCR as described elsewhere (Datsenko and Wanner, 2000).

Plasmids pUH*pmrAB* containing the H35A and H57A substitutions were constructed using the QuickChange II Site-directed Mutagenesis Kit (Stratagene) with primers 7075 (5′-AGTACCTTCTGGTTATGGGCTGAAAGCACTGAGCA-3′) and 7076 (5′-TGCTCAGTGCTTTCAGCCCATAACCAGAAGGTACT-3′); 7077 (5′-AATCGCAACAACGATCGCGCTATCATGCACGAAAT-3′) and 7078 (5′-ATTTCGTGCATGATAGCGCGATCGTTGTTGCGATT-3′) respectively.

### β-Galactosidase assays

Cells were grown overnight in N-minimal media, pH 7.7, and washed once in N-minimal media pH 7.7 or 5.8 before inoculation into media of the same pH. Activity was determined as described elsewhere (Miller, 1972) after 4 h of growth at 37°C.

### Immunoblotting analysis

Cells were grown in 20 ml of N-minimal media, pH 7.7 or 5.8, to OD_600_∼0.5, washed with TBS twice, resuspended in 500 μl of TBS and opened by sonication. Whole-cell lysates were run on NuPAGE Bis-Tris gels (Invitrogen) with MES running buffer, transferred to PVDF membranes and analysed by immunoblotting with an anti-PmrD polyclonal antibody. Blots were developed by using anti-rabbit IgG horseradish peroxidase-linked antibodies (Amersham Biosciences) and Supersignal West Femto (Pierce).

### RNA isolation, reverse transcription-PCR (RT-PCR) and real-time PCR

Cells were grown in 10 ml of N-minimal media, pH 7.7 or 5.8, to OD_600_∼0.5. One millilitre of culture was used to prepare total RNA using the SV Total RNA Isolation System (Promega). cDNA was synthesized using TaqMan (Applied Biosystems) and random hexamers following the manufacturer's instructions. Quantification of transcripts was performed by real-time PCR using SYBR Green PCR Master Mix (Applied Biosystems) in an ABI 7000 Sequence Detection System (Applied Biosystems). Two different sets of primers were used to detect the *pmrD* transcript (both gave similar results): 4491 (5′-GGTTAAGAAATCGCATTATGTCAAAA-3′) and 4492 (5′-CGAACCGCCGCTATCG-3′); 6528 (5′-TGGAATGGTTGGTTAAGAAATCG-3′) and 6529 (5′-CATGGCACGCCCTCTTTTT-3′). Primers 6496 (5′-AGCGATAGGCATTGAGCAGC-3′) and 6497 (5′-CAGGTTTGCCGCGAAATTAG-3′) were used to detect the *slyA* transcript and 6213 (5′-GCTGGAAGTCGAGGAGTCACA-3′) and 6214 (5′-TCGTCCGGTTCGACCAAA-3′) to quantify the *corA* transcript. Results were normalized to the levels of 16S ribosomal RNA which were estimated using primers 3032 (5′-CCAGCAGCCGCGGTAAT-3′) and 3034 (5′-TTTACGCCCAGTAATTCCGATT-3′). The amount of each PCR product was calculated from standard curves obtained from PCR with the same primers and serially diluted DNA.

### Polymyxin B susceptibility assay

Assays were performed following a previously described protocol (Groisman *et al*., 1992) with a few modifications. Bacteria were grown overnight in N-minimal media, pH 7.7, containing 10 mM MgCl_2_, and washed once in N-minimal media pH 7.7 or 5.8 before inoculation (1:50 dilution) into 10 ml of media of the same pH. Cells were grown at 37°C with aeration to OD_600_∼0.6 and diluted 1:100 in LB broth. A 300 μg ml^−1^ stock solution of water-dissolved polymyxin B was diluted 1:100 in LB broth immediately before the assay. Fifty microlitres of diluted cells and 50 μl of diluted polymyxin B solution were mixed and placed in 96-well plates for 1 h at 37°C with shaking. A portion of each sample was serially diluted and plated on LB agar plates to determine the number of colony-forming units (cfu). Per cent survival was calculated as follows: